# DNTGF-βR armored CAR-T cell therapy against tumors from bench to bedside

**DOI:** 10.1186/s12967-023-04829-6

**Published:** 2024-01-11

**Authors:** Yuning Wang, Guo Zhao, Shuhang Wang, Ning Li

**Affiliations:** Clinical Trial Center, National Cancer Center/National Clinical Research Center for Cancer/Cancer Hospital, Chinese Academy of Medical Sciences and Peking Union Medical College, Beijing, 100021 China

**To the editor,**

CAR-T cell therapy has achieved great success in hematological malignancies, but its effectiveness in solid tumors has been limited due to the complicated immunosuppressive tumor microenvironment (TME). TGF-β, a negative cytokine, exerts critical roles in shaping immunosuppressive TME, thereby promoting tumor progression and resistance [1]. Thus, evolving approaches to blocking TGF-β signaling in CAR-T cell therapy have been emerging, such as combining with TGF-β-targeted neutralizing antibodies or small molecule inhibitors, directly deleting TGF-βRII via CRISPR/Cas9 technology, or co-expressing a dominant-negative TGF-β receptor II (DNTGF-βRII). As a relatively niche modification strategy, dominant negative receptor (DNR) technology has received less attention. However, its potential translational value warrants further underscore.

## Distribution landscape of DNR armored CAR-T cell therapy in cancer research

**Fig. 1:**
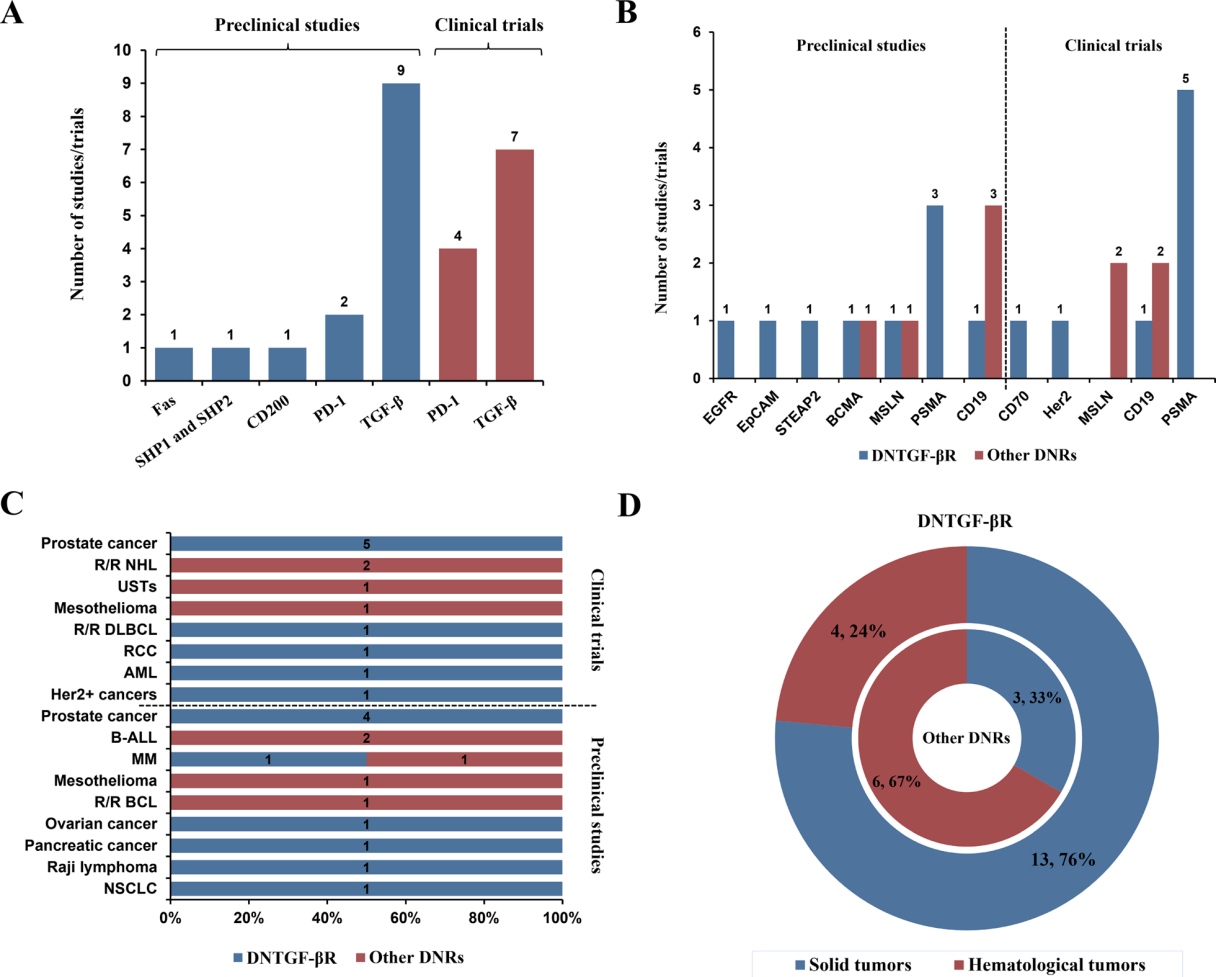
Distribution landscape of DNR armored CAR-T cell therapy in cancer research.  **A** Distribution of DNRs in preclinical studies and clinical trials;  **B **Comparison between DNTGF-βR and other DNRs in CAR targets;  **C** Comparison between DNTGF-βR and other DNRs in specific cancer types;  **D** Comparison between DNTGF-βR and other DNRs in proportions of cancer types (solid tumors/hematological tumors)

To provide a comprehensive understanding of current status of DNR armored CAR-T cell therapy against tumors, we systematically investigated all relevant preclinical studies and clinical trials to assess its distribution and demonstrate its translational potential. As shown in Fig. 1A, preclinical studies obviously had a broader DNR spectrum, among which DNTGF-βR and PD-1 DNR ranked the top 2, along with some previously unexplored targets such as Fas, SHP1 and SHP2, and CD200. However, only DNTGF-βR and PD-1 DNR entered early trial phases currently.

For CAR targets and cancer types, significant discrepancies could also be observed between DNTGF-βR and other DNRs armored CAR-T cell therapies, preclinical studies and clinical trials (Fig. 1B, C and D, Additional file 1: Table S1, Additional file 2: Table S2, S3). For example, trials of DNTGF-βR armored CAR-T therapy commonly targeted PSMA-expressing metastatic castration resistant prostate cancer (mCRPC) (Fig. 1B and C, Additional file 2: Table S2). While PD-1 DNR armored CAR-T cell therapies focused more on CD19-expressing relapsed/ refractory Non-Hodgkin Lymphoma (r/r NHL) and MSLN-expressing tumors such as mesothelioma (Fig. 1B and C, Additional file 2: Table S3). By contrast, preclinical studies of DNTGF-βR armored CAR-T cell therapy took more tries on new targets and different indications, such as a novel target STEAP2 overexpressed in prostate cancer, or MSLN-expressing ovarian cancer (Fig. 1B and C, Additional file 2: Table S2). Moreover, solid tumors were ideal indications for DNTGF-βR armored CAR-T cell therapy due to the crucial negative role of TGF-β in TME (Fig. 1D). All of these indicated DNTGF-βR was one of the most mature intrinsic genetic engineering strategies in CAR-T cell therapy.

### Evidence of efficacy and safety on DNTGF-βR armored CAR-T cell therapy in preclinical studies/clinical trials

Actually, DNTGF-βR armored CAR-T cell therapy could be traced back to 2009, a trial (NCT00889954) relevant to Her2 CAR and TGF-β DNR expressing EBV specific lymphocytes against Her2 positive malignancies, but no results were reported so far. Back to 2018, Carl H June’s team successfully constructed DNTGF-βRII armored CAR-T cells, which exhibited enhanced proliferation and cytokine secretion level, endowed with exhaustion-resistant and long-term in vivo persistent properties, and induced tumor regression in prostate cancer models [2]. These positive preclinical results prompted an initiation of a first-in-human phase I clinical trial (NCT03089203). In this trial, primary endpoints of safety and feasibility were met. For clinical outcomes, 4 of 13 patients achieved reductions in PSA levels ≥ 30%, and tumor eradication was observed in one patient, whose median overall survival (mOS) was 15.9 months [3]. Beyond the only published clinical trial results, some other clinical trials investigating DNTGF-βR armored CAR-T cell therapy are open for recruiting (ChiCTR1900024218) or being planned (NCT06046040, TrialTroveID-485361). Notably, one of the trials on testing a universal DNTGF-βR armored CD70 CAR γδ T cell therapy is under planning, deserving close attention. Furthermore, increasing preclinical data showcased considerably superior efficacy and safety across different CAR targets/indications. Especially in a recent study, another DNTGF-βRII-armored CAR-T cell therapy, AZD0754, demonstrated its exceptional antitumor activity and encouraging safety in STEAP2-expressing prostate cancer [4]. STEAP2 as a novel target was never investigated in previous CAR-T cell therapies. This study emphasized the therapeutic potential of STEAP2 and further promoted the development of DNTGF-βRII-armored CAR-T cell therapy.

### Head-to-head comparisons between DNR technology and other signaling blockade strategies

In many studies, researchers have tried to confirm and explain the advantages of this technology over other signaling blockade strategies. In comparison to TGF-βRII deletion, DNTGF-βRII conferred CAR-T cells with a more powerful proliferation potential despite the presence of residual TGF-β signaling [3]. In another study, the researchers found PD1 DNR was more dependable than shRNA-mediated PD-1 knockdown, and assumed a conclusion that PD1 DNR or PD-1 antibody (comparison between the two) was more superior in efficacy could not be drawn when a range of potential influencing variables were considered [5]. But to some extent, single administration of PD-1 DNR CAR-T cells provided much more convenience than repeated antibody administrations and might reduce the incidence of immune-related adverse events (IRAEs). Additionally, a recent study reported that a CD200R-CD28 switch optimally outperformed in enhancing CAR-T function in CD200 + multiple myeloma (MM) models than the approach of CD200R-dominant negative [6]. And surprisingly, the CD200RKO unexpectedly reduced cytotoxicity conversely. These suggested CD200R checkpoint receptor might exert positive roles in affecting T cell function in specific contexts. Generally, there is no definite conclusion of differentiation at present, and more research needs to be implemented under different conditions.

## Conclusions

In conclusion, DNTGF-βR entrusted CAR-T cells with a new capability to circumvent immunosuppressive TME so as to improve antitumor efficacy. Although, potential risks and challenges still remain. Most importantly, novel targets are urgent to be discovered and innovative genetic engineering strategies should be encouraged to investigate to promote their translations from bench to bedside.

### Supplementary Information

** Additional file 1: Table S1**. Detailed information of clinical trials of DNR armored CAR-T cell therapy in cancer research (Clinical trial data from Informa database).

**Additional file 2:**  **Table S2. **Summary of preclinical studies/clinical trials relevant to DNTGF-βR armored CAR-T cell therapy against tumors (Clinical trial data from Informa database and clinicaltrials.gov). **Table S3. **Summary of preclinical studies/clinical trials relevant to dominant negative receptor (DNR) (except TGF-βRII) armored CAR-T cell therapy (Clinical trial data from Informa database and clinicaltrials.gov).

## Data Availability

All data for this study are publicly available.
