# Supplementary material for: DNTGF-βR armored CAR-T cell therapy against tumors from bench to bedside
Source: J Transl Med. 2024 Jan 11;22:45. doi: 10.1186/s12967-023-04829-6 (PMC10782706; doi:10.1186/s12967-023-04829-6)
Supplement: Supplementary file 2 — Additional file 2: Table S2. Summary of preclinical studies/clinical trials relevant to DNTGF-βR armored CAR-T cell therapy against tumors (Clinical trial data from Informa database and clinicaltrials.gov). Table S3. Summary of preclinical studies/clinical trials relevant to dominant negative receptor (DNR) (except TGF-βRII) armored CAR-T cell therapy (Clinical trial data from Informa database and clinicaltrials.gov). [file 12967_2023_4829_MOESM2_ESM.docx]

**Additional files**

**DNTGF-βR armored CAR-T cell therapy against tumors** **from bench to bedside**

**Authors:** Yuning Wang^1,*^, Guo Zhao^1,*^, Shuhang Wang^1,#^, Ning Li^1,#^

^1^Clinical Trial Center, National Cancer Center/National Clinical Research Center for Cancer/Cancer Hospital, Chinese Academy of Medical Sciences and Peking Union Medical College, Beijing 100021, China.

*These authors contributed equally to this work.

#Corresponding authors.

E-mail addresses: snowflake201@gmail.com (S. Wang), lining@cicams.ac.cn (N. Li)

**Table S2. Summary of preclinical studies/clinical trials relevant to DNTGF-βR armored CAR-T cell therapy against tumors (Clinical trial data from Informa database and clinicaltrials.gov).**

| **PMID or Trial ID** | **Phase (Status)** | **Primary drug** | **CAR target** | **Cancer type** | **Publication year/Trial start year** |
| --- | --- | --- | --- | --- | --- |
| **Preclinical studies** | | | | |  |
| 29275833 | Preclinical | / | PSMA | mCRPC | 2018 |
| 29807781 | Preclinical | / | PSMA | Prostate cancer | 2018 |
| 34108220 | Preclinical | / | EpCAM | Pancreatic cancer | 2021 |
| 33974997 | Preclinical | / | CD19 | Raji lymphoma | 2021 |
| 35222421 | Preclinical | / | BCMA | MM | 2022 |
| 36166071 | Preclinical | / | MSLN | Ovarian cancer | 2023 |
| 37376985 | Preclinical | / | EGFR- | NSCLC | 2023 |
| 37690238 | Preclinical | / | PSMA | Prostate cancer | 2023 |
| 37966111 | Preclinical | AZD0754 | STEAP2 | mCRPC | 2023 |
| **Clinical trials** | | | | |  |
| NCT00889954 | I (Completed) | AU-105 | Her2 | HER2 positive malignancies | 2009 |
| 35314843;  NCT03089203 | I (Open) | TmPSMA-01 | PSMA | mCRPC | 2022;  2017 |
| ChiCTR1900024218 | I (Open) | CAR-T-19-DNR, Immunotech Applied Science | CD19 | R/R DLBCL | 2019 |
| NCT04227275 | I (Terminated) | TmPSMA-01 | PSMA | mCRPC | 2019 |
| NCT05489991 | I/Ⅱ (Terminated) | TmPSMA-02 | PSMA | mCRPC | 2022 |
| NCT06046040 | I (Planned) | TmPSMA-02 | PSMA | mCRPC | 2024 |
| TrialTroveID-485361 | I (Planned) | ADI-270 | CD70; PSMA | AML and RCC; mCRPC | / |

**Abbreviations:** CAR, chimeric antigen receptor; PSMA, prostate-specific membrane antigen; mCRPC, metastatic castration resistant prostate cancer; EpCAM, epithelial cell adhesion molecule; BCMA, B-cell maturation antigen; MM, multiple myeloma; MSLN, mesothelin; EGFR, epidermal growth factor receptor; NSCLC, non-small cell lung cancer; STEAP2, six-transmembrane epithelial antigen of prostate-2; Her2, epidermal growth factor receptor 2; DNR, dominant negative receptor; R/R DLBCL, relapsed/refractory diffuse large b-cell lymphoma; AML, acute myeloid leukemia; RCC, renal cell carcinoma.

**Table S3. Summary of preclinical studies/clinical trials relevant to dominant negative receptor (DNR) (except TGF-βRⅡ) armored CAR-T cell therapy** **(Clinical trial data from Informa database and clinicaltrials.gov).**

| **PMID or Trial ID** | **Phase (Status)** | **Primary drug** | **DNR** | **CAR target** | **Cancer type** | **Publication year/Trial start year** |
| --- | --- | --- | --- | --- | --- | --- |
| **Preclinical trials** | | | | | |  |
| 27454297 | Preclinical | / | PD-1 | MSLN | Pleural mesothelioma | 2016 |
| 30694219 | Preclinical | / | Fas | CD19 | B-ALL | 2019 |
| 33813229 | Preclinical | / | PD-1 | CD19 | R/R BCL | 2021 |
| 37334382 | Preclinical | / | SHP1 and SHP2 | CD19 | B-ALL | 2023 |
| 37616575 | Preclinical | / | CD200 | BCMA | MM | 2023 |
| **Clinical trials** | | | | | |  |
| ChiCTR1900021295 | I/Ⅱ (Completed) | ICTCAR-014 | PD-1 | CD19 | R/R NHL | 2018 |
| TrialTroveID-362825 | I (Terminated) | ICTCAR-014 | PD-1 | CD19 | R/R NHL | 2020 |
| NCT04577326 | I (Open) | ATA-2271 | PD-1 | MSLN | Mesothelioma | 2020 |
| TrialTroveID-367895 | Ⅱ (Planned) | M28zPD1DNR CAR T cell | PD-1 | MSLN | USTs | 2020 |

**Abbreviations:** DNR, dominant negative receptor; CAR, chimeric antigen receptor; PD-1, programmed cell death protein 1; MSLN, mesothelin; B-ALL, B cell acute lymphoblastic leukemia; R/R BCL, refractory/relapsed B cell lymphoma; BCMA, B-cell maturation antigen; MM, multiple myeloma; R/R NHL, relapsed or refractory Non-Hodgkin Lymphoma; USTs, unspecified solid tumors.
